# Robotic-assisted unicompartmental knee arthroplasty is associated with lower odds of prolonged hospitalization and no higher odds of high-charge admission during the index hospitalization

**DOI:** 10.1007/s00402-026-06336-x

**Published:** 2026-05-09

**Authors:** Qiaoqiao Sun, Ying Xu, Xiaodan Hong, Ming Dai, Jianping Wang, Hao Xie, Zicai Fu

**Affiliations:** 1 School of Management, Guangdong University of Technology, Guangzhou, China; 2 Innovation Theory and Innovation Management Research Center, Guangdong University of Technology, Guangzhou, China; 3 Division of Orthopaedic Surgery, Department of Orthopaedics, Nanfang Hospital, Southern Medical University, Guangzhou, China; 4 Department of Neurology, Nanfang Hospital, Southern Medical University, Guangzhou, China; 5 Queen Mary School, Nanchang University, Nanchang, China; 6 Department of Medical Imaging Center, Nanfang Hospital, Southern Medical University, Guangzhou, China; 7 Department of Orthopedics, Southern Medical University Pingshan Hospital, Shenzhen, China

**Keywords:** Robotic-assisted surgery, Unicompartmental knee arthroplasty, Prolonged hospitalization, Billed hospital charges, National inpatient sample

## Abstract

**Background:**

Robotic-assisted unicompartmental knee arthroplasty (RA-UKA) may improve implant positioning, but its impact on index-hospitalization length of stay (LOS), billed charges, and early inpatient outcomes in routine practice remains unclear.

**Methods:**

Using the National Inpatient Sample (2016–2022), we identified primary medial UKA for osteoarthritis. RA-UKA was defined by ICD-10-PCS robotic-assisted lower-extremity procedure codes. Primary outcomes were prolonged hospitalization (LOS ≥ the 75th percentile, ≥ 2 days), high-charge admission (total hospital charges [TOTCHG] ≥ the 75th percentile, ≥ $72,321), and in-hospital mortality. We performed univariable comparisons and multivariable logistic regression adjusting for demographics, payer, admission type, hospital teaching status, comorbidities, and calendar year fixed effects (YEAR 2016–2022) using the unweighted NIS discharge sample.

**Results:**

Among 7,154 UKAs, 1,297 (18.1%) were RA-UKA and 5,857 (81.9%) were C-UKA. Median LOS was 1 day (IQR 1–2) in both groups; however, prolonged hospitalization (LOS ≥ 2 days) occurred less frequently in RA-UKA (412/1,297 [30.7%] vs. 2,162/5,857 [37.2%]; *P* < 0.001). In adjusted analyses including YEAR fixed effects, RA-UKA was associated with lower odds of prolonged hospitalization (aOR 0.750, 95% CI 0.647–0.870; *P* < 0.001) and was not associated with high-charge admission (TOTCHG ≥ $72,321; aOR 1.007, 95% CI 0.855–1.186; *P* = 0.993). In-hospital mortality and other inpatient complications were rare.

**Conclusions:**

RA-UKA was associated with lower odds of prolonged hospitalization (LOS ≥ 2 days) and no higher odds of a high-charge admission (billed charges, TOTCHG) during the index hospitalization. Findings apply to the index hospitalization only.

**Supplementary Information:**

The online version contains supplementary material available at 10.1007/s00402-026-06336-x.

## Introduction

 Unicompartmental knee arthroplasty (UKA) provides a surgical option that is less invasive than total knee arthroplasty (TKA) for the management of single-compartment knee osteoarthritis [1]. This procedure conserves native bone and cruciate ligaments, which is associated with quicker rehabilitation and reduced perioperative morbidity relative to TKA [2, 3]. However, UKA remains a minority procedure relative to total knee arthroplasty in contemporary practice [4]. Concerns regarding revision risk and the technical demands of UKA have been cited as barriers, motivating interest in technologies such as robotic assistance to improve reproducibility and reduce technical variability [5, 6].

Over the last ten years, the introduction of robotic-assisted systems has been a key technological advance intended to increase the reproducibility and accuracy of UKA, thereby potentially mitigating modes of failure. Platforms for robotic-assisted UKA (RA-UKA), including MAKO and NAVIO, facilitate improved component positioning and soft-tissue balancing compared to manual methods [7, 8]. Initial clinical evaluations suggest that RA-UKA may improve radiographic implant alignment and reduce positioning outliers [9, 10]. However, whether these technical advantages translate into improved inpatient outcomes in routine practice remains uncertain [11, 12]. Despite this promise, robust evidence demonstrating these advantages remains scarce. The existing literature on RA-UKA consists predominantly of small, single-center series or cohort studies from specialized institutions [7, 8], and reported clinical outcomes have been inconsistent [13].

Conversely, RA-UKA implementation incurs additional costs related to robotic technology and may be associated with increased operative time [14, 15]. While cost-effectiveness discussions often emphasize long-term revision risk and functional outcomes [16], inpatient endpoints such as LOS and billed charges reflect perioperative efficiency and resource use during the index hospitalization and deserve separate evaluation. Notably, in the NIS, total hospital charges (TOTCHG) represent billed charges rather than true costs or reimbursements.

This study was designed to address these evidence gaps by employing a comprehensive US inpatient database to compare inpatient outcomes between RA-UKA and C-UKA. National datasets such as the NIS are valuable for assessing rare complications and capturing nationwide utilization patterns outside of tertiary referral centers [17]. Importantly, inpatient outcomes such as length of stay and billed hospital charges reflect perioperative efficiency and resource use and therefore warrant evaluation independent of long-term revision risk or patient-reported outcomes, which are not captured in administrative inpatient datasets. Because LOS and charges capture different constructs, we anticipated a more direct association with prolonged LOS than with billed charges, which may be offset by technology-related billing. We hypothesized that RA-UKA would be associated with lower odds of prolonged hospitalization and similar early in-hospital complication rates compared with C-UKA, without higher odds of a high-charge admission during the index hospitalization.

## Materials and methods

### Database source

This retrospective analysis used the National Inpatient Sample (formerly the Nationwide Inpatient Sample; NIS) from 2016 to 2022, maintained by the Healthcare Cost and Utilization Project (HCUP). Beginning in 2012, the NIS approximates a 20% stratified sample of discharges from U.S. community hospitals and includes discharge weights that can be used to generate national estimates [18]. The data encompass patient demographics, hospital characteristics, and up to 40 diagnosis and procedure codes (ICD-10-CM/PCS). The use of this de-identified, publicly available dataset granted an exemption from institutional review board oversight.

### Cohort identification and exclusions

The initial study population was extracted from the NIS by identifying all hospital discharges from 2016 to 2022 that contained relevant procedure codes for UKA. The identification of medial UKA procedures relied on specific ICD-10-PCS codes, including 0SRC0L9, 0SRC0LA, 0SRC0LZ, 0SRD0L9, 0SRD0LA, and 0SRD0LZ [19].

A hospitalization was categorized as a RA-UKA if the record additionally included codes designating robotic-assisted lower-extremity procedures, specifically 8E0Y0CZ and 8E0YXCZ [20]. All other UKA cases, lacking these specific robotic modifiers, were defined as C-UKA. Robotic assistance was identified using ICD-10 procedure codes; misclassification due to coding inaccuracies is possible.

To establish a homogeneous study group, we included primary medial UKA procedures performed for a diagnosis of osteoarthritis. Exclusions applied to lateral compartment UKAs, revision arthroplasty procedures, and patients under the age of 18. To uphold data integrity, we also removed any entries with incomplete information for critical fields, such as age, sex, length of stay, and total hospital charges.

After applying these criteria, the final analytical dataset included 7,154 UKA cases from an initial pool of 9373 hospitalizations. This comprised 1297 (18.1%) RA-UKA procedures and 5,857 (81.9%) C-UKA procedures.

### Variables and outcomes

The data captured for each patient encompassed demographic details (age, sex, race [White, Black, Hispanic, Other]), and the primary insurance payer (Medicare, Medicaid, private, or other). Hospital-level information included teaching status. We ascertained comorbid conditions from ICD-10 diagnosis codes, which comprised: AIDS/HIV, alcohol abuse, chronic blood loss anemia, coagulopathy, dementia, hypertension, autoimmune disorders, deficiency anemias, depression, drug abuse, obesity, paralysis, peripheral vascular disease, weight loss, dyslipidemia, obstructive sleep apnea, osteoporosis, mental health conditions, Parkinson disease, type 2 diabetes, chronic pulmonary disease, and chronic renal disease. The admission was also characterized as either elective or non-elective.

The principal study endpoints were hospital LOS, total hospital charges, the occurrence of postoperative complications, and mortality during the hospitalization. LOS was quantified in days, and hospital charge data were acquired directly from the NIS. Total hospital charges (TOTCHG) reflect billed charges and do not represent hospital costs or reimbursements. Prolonged hospitalization was defined as LOS ≥ the 75th percentile in the analytic cohort (≥ 2 days). High-charge admission was defined as TOTCHG ≥ the 75th percentile (≥ $72,321). Charges were analyzed in nominal U.S. dollars as reported by NIS without inflation adjustment; calendar year fixed effects were included in all models to account for secular trends. We selected the 75th percentile to identify upper-tail, resource-intensive utilization while maintaining sufficient event counts for stable modeling, given the right-skewed distributions of LOS and hospital charges.

The specific postoperative complications evaluated included acute kidney injury (AKI), acute coronary syndrome, cerebrovascular accident (stroke), pneumonia, pulmonary embolism (PE), deep vein thrombosis (DVT), anemia due to blood loss, acute heart failure and congestive heart failure. Each complication, along with in-hospital mortality, was evaluated as a binary (yes/no) outcome. Because the NIS is an inpatient database, it does not provide information on long-term outcomes such as hospital readmission or subsequent revision surgery.

### Statistical analysis

Baseline demographic and clinical characteristics were compared between the RA-UKA and C-UKA groups. Categorical variables were analyzed using chi-square tests, and Fisher’s exact tests were applied when expected cell counts were < 5. Continuous variables were non-normally distributed and were therefore compared using the Wilcoxon rank-sum test. These univariable analyses were used to describe crude differences between surgical approaches. A two-sided P value < 0.05 was considered statistically significant.

Multivariable logistic regression was employed as the principal method of adjustment. Separate models were constructed for each outcome, with surgical technique (RA-UKA vs. C-UKA) as the primary exposure. Regression models were adjusted for clinically relevant covariates selected a priori, including age, sex, race, primary payer, admission type (elective vs. non-elective), hospital teaching status, the comorbid conditions listed in Table 1, and calendar year fixed effects (YEAR 2016–2022) to account for potential secular trends across the study period.

Multicollinearity among covariates was assessed using collinearity diagnostics (tolerance and variance inflation factors), and no concerning multicollinearity was identified (all VIFs < 2). Because the primary purpose of the models was covariate adjustment rather than prediction, model discrimination metrics (e.g., C-statistics) were not emphasized. Race data were missing in 314 of 9373 discharges (3.4%), and these cases were excluded from multivariable analyses (complete-case analysis). Comorbidities were entered as individual covariates rather than a composite index to preserve clinical interpretability and to account for potentially differential associations of specific conditions with LOS and in-hospital outcomes.

Results are reported as adjusted odds ratios (aORs) with 95% confidence intervals (CIs). For extremely sparse outcomes, conventional logistic regression may fail to converge because of separation; in such cases, adjusted estimates were considered non-estimable and were interpreted descriptively rather than as evidence of equivalence. Penalized (Firth) or exact logistic regression was not pursued because event counts were too low to support stable covariate-adjusted inference in this administrative dataset, and our primary inference focused on LOS- and charge-related endpoints. Analyses were conducted on the unweighted discharge-level sample (no NIS sampling weights applied), as our primary aim was to estimate adjusted associations within the analytic cohort rather than generate nationally weighted incidence estimates. All analyses were conducted using SPSS (version 25; IBM Corp., Armonk, NY).

## Results

### Patient cohort and distribution of surgical approach

A total of 7,154 UKA procedures were included in the analysis. RA-UKA accounted for 18.1% of all cases, whereas C-UKA represented 81.9% of procedures (Table 1). Age was similar between groups (66.0 [IQR 60.0–73.0] in the RA-UKA group vs. 66.0 [IQR 60.0–72.0] in the C-UKA group; *P* = 0.124), and the proportion of female patients did not differ significantly (51.3% vs. 50.6%, *P* = 0.644). Race data were missing in 314 of 9,373 discharges (3.4%); these cases were excluded from multivariable analyses.

There was a significant difference in racial distribution between the two groups (*P* < 0.001). Patients undergoing RA-UKA had a lower proportion of White (82.2% vs. 84.2%) and Black patients (4.8% vs. 5.6%), and a higher proportion of Hispanic patients (9.0% vs. 5.7%) compared with those undergoing C-UKA (Table 1). Primary expected payer was comparable between RA-UKA and C-UKA (Medicare 42.2% vs. 42.0%, Medicaid 5.1% vs. 5.3%, private 46.9% vs. 47.2%, other 5.9% vs. 5.5%; overall *P* = 0.955). The majority of procedures in both groups were elective (94.7% vs. 94.6%, *P* = 0.875).

Regarding hospital characteristics, teaching status differed significantly between groups: RA-UKA was performed in teaching hospitals in 54.8% of cases, compared with 61.8% for C-UKA (*P* < 0.001), indicating a tendency for C-UKA to be more frequently performed in teaching institutions. These differences may reflect variation in access to robotic technology and institutional practice patterns.

### Comorbidities

Overall comorbidity profiles were similar between groups, with only a few statistically significant differences (Table 1). Compared with C-UKA, the RA-UKA cohort had lower rates of deficiency anemia (2.9% vs. 4.4%, *P* = 0.016), coagulopathy (1.4% vs. 2.3%, *P* = 0.042), mental disorders (25.1% vs. 28.7%, *P* = 0.009), and drug abuse (0.2% vs. 0.6%, *P* = 0.035), and a slightly higher prevalence of dementia (0.9% vs. 0.5%, *P* = 0.040). All other assessed comorbidities were comparable between groups (all *P* > 0.05).

**Table 1:** Baseline demographic and clinical characteristics of patients undergoing UKA

Variable	RA-UKA (*n* = 1297)	C-UKA (*n* = 5857)	*P*
Population demographics			
Total cases, n (%)	1,297 (18.1)	5,857 (81.9)	
Age, years, median [IQR]	66.0 [60.0–73.0]	66.0 [60.0–72.0]	0.124
Female, n (%)	665 (51.3)	2,964 (50.6)	0.644
Race, n (%)			< 0.001
White	1,066 (82.2)	4,932 (84.2)	
Black	62 (4.8)	328 (5.6)	
Hispanic	117 (9.0)	334 (5.7)	
Other	52 (4.0)	264 (4.5)	
Primary expected payer, n (%)			0.955
Medicare	547 (42.2)	2,460 (42.0)	
Medicaid	66 (5.1)	310 (5.3)	
Private	608 (46.9)	2,765 (47.2)	
Other	77 (5.9)	322 (5.5)	
Elective, n (%)	1,228 (94.7)	5,541 (94.6)	0.875
Hospital characteristics, n (%)			
Teaching hospital	711 (54.8)	3,620 (61.8)	< 0.001
Comorbidities			
Cardiovascular, n (%)			
Hypertension	663 (51.1)	3,087 (52.7)	0.295
Peripheral vascular disease	23 (1.8)	135 (2.3)	0.238
Dyslipidemia	521 (40.2)	2,519 (43.0)	0.064
Obesity	316 (24.4)	1,564 (26.7)	0.083
Endocrine/metabolic, n (%)			
Type 2 diabetes mellitus	224 (17.3)	978 (16.7)	0.551
Weight loss	3 (0.2)	12 (0.2)	0.999
Deficiency anemias	38 (2.9)	258 (4.4)	0.016
Respiratory, n (%)			
Chronic lung disease	52 (4.0)	275 (4.7)	0.525
Obstructive sleep apnea	228 (17.6)	978 (16.7)	0.425
Renal, n (%)			
Chronic kidney disease	58 (4.5)	299 (5.1)	0.319
Hematologic/immune, n (%)			
Coagulopathy	18 (1.4)	135 (2.3)	0.042
Autoimmune condition	30 (2.3)	152 (2.6)	0.515
Neurologic/psychiatric, n (%)			
Dementia	12 (0.9)	29 (0.5)	0.040
Depression	147 (11.3)	726 (12.4)	0.264
Mental disorders	326 (25.1)	1,681 (28.7)	0.009
Parkinson disease	6 (0.5)	23 (0.4)	0.582
Substance-related disorders, n (%)			
Alcohol abuse	3 (0.2)	29 (0.5)	0.247
Drug abuse	3 (0.2)	35 (0.6)	0.035
Infectious disease, n (%)			
AIDS	3 (0.2)	6 (0.1)	0.214
Others, n (%)			
Osteoporosis	36 (2.8)	135 (2.3)	0.335
Paralysis	6 (0.5)	23 (0.4)	0.519
Chronic blood loss anemia	1 (0.1)	12 (0.2)	0.706

Age is presented as median [IQR]. All other values are presented as n (%). P values were calculated using chi-square tests or Fisher’s exact tests for categorical variables, as appropriate, and the Wilcoxon rank-sum test for age. *RA-UKA* robotic-assisted unicompartmental knee arthroplasty, *C-UKA* conventional unicompartmental knee arthroplasty, *IQR* interquartile range

### Primary outcomes: length of stay, total charges, and in-hospital mortality

 Median LOS was 1 day (IQR 1–2) in both groups. Because the 75th percentile for LOS in the analytic cohort was 2 days, prolonged hospitalization (LOS ≥ 2 days) captures the upper tail of the LOS distribution and occurred less frequently in RA-UKA (412/1,297 [30.7%]) than in C-UKA (2,162/5,857 [37.2%]; *P* < 0.001). Median total charges (TOTCHG) were similar between groups (RA-UKA: $49,368 [IQR $36,186–$71,616] vs. C-UKA: $47,756 [IQR $34,495–$70,394]; *P* = 0.083).

In adjusted analyses including YEAR fixed effects (2016–2022), RA-UKA was associated with lower odds of prolonged hospitalization (aOR 0.750, 95% CI 0.647–0.870; *P* < 0.001). High-charge admission (TOTCHG ≥ $72,321) occurred in 323/1,297 (24.9%) RA-UKA versus 1,467/5,857 (25.0%) C-UKA admissions, and RA-UKA was not associated with high-charge admission after adjustment (aOR 1.007, 95% CI 0.855–1.186; *P* = 0.993). In-hospital mortality and other inpatient complications were rare.

**Table 2:** Univariable and multivariable analyses of primary outcomes after UKA

Outcome	RA-UKA (*n* = 1297)	C-UKA (*n* = 5857)	Unadjusted *P*	Adjusted aOR	95% CI	Adjusted *P*
LOS, days (median [IQR])	1 [1–2]	1 [1–2]	–	–	–	–
Prolonged hospitalization (LOS ≥ 2 days), n (%)	412 (30.7)	2,162 (37.2)	< 0.001	0.750	0.647–0.870	< 0.001
Total charges (TOTCHG), USD (median [IQR])	49,368 [36,186–71,616]	47,756 [34,495–70,394]	0.083	–	–	–
High-charge admission (TOTCHG ≥ $72,321), n (%)	323 (24.9)	1,467 (25.0)	0.353	1.007	0.855–1.186	0.993
In-hospital mortality, n (%)	0 (0.0)	1 (0.02)	0.999	–	–	–

LOS and TOTCHG are presented as median (IQR). Prolonged hospitalization was defined as LOS ≥ the 75th percentile (≥ 2 days). High-charge admission was defined as TOTCHG ≥ the 75th percentile (≥ $72,321). Unadjusted P values were calculated using the Wilcoxon rank-sum test for continuous variables and chi-square or Fisher’s exact tests for categorical variables, as appropriate. Adjusted odds ratios (aORs) and 95% confidence intervals (CIs) were estimated using multivariable logistic regression adjusting for age, sex, race, primary payer, admission type, hospital teaching status, comorbidities, and calendar year fixed effects (YEAR 2016–2022). TOTCHG reflects billed hospital charges and does not represent costs or reimbursements. In-hospital mortality was summarized descriptively because of sparse events and the lack of stable adjusted estimation. *RA-UKA* robotic-assisted unicompartmental knee arthroplasty, *C-UKA* conventional unicompartmental knee arthroplasty, *LOS* length of stay, *TOTCHG* total hospital charges, *aOR* adjusted odds ratio, *CI* confidence interval

### Medical complications

In the univariable analysis, the incidence of medical complications—including acute kidney injury (AKI), stroke, pneumonia, congestive heart failure, and acute heart failure—did not differ significantly between RA-UKA and C-UKA (all *P* > 0.05; Table 3).

Adjusted associations between surgical approach and major medical complications are summarized in Table 3. RA-UKA was not significantly associated with AKI (aOR 0.743, 95% CI 0.389–1.419; *P* = 0.369), congestive heart failure (aOR 1.287, 95% CI 0.657–2.523; *P* = 0.462), or acute heart failure (aOR 1.244, 95% CI 0.791–1.955; *P* = 0.344) compared with C-UKA.

For stroke and pneumonia, logistic regression models failed to converge because of extremely low event frequencies, and adjusted aORs and 95% CIs were therefore not estimable. The P values from the Score test were 0.996 for stroke and 0.988 for pneumonia, indicating no statistically detectable differences between RA-UKA and C-UKA within the constraints of the available data. As with mortality, these findings primarily reflect insufficient events rather than evidence of equivalence.

**Table 3:** Univariable and multivariable analyses of medical complications after UKA

Variable	RA-UKA (*n* = 1297)	C-UKA (*n* = 5857)	Unadjusted *P*	Adjusted aOR	95% CI	Adjusted *P*
AKI	12 (0.9)	78 (1.3)	0.235	0.743	0.389–1.419	0.369
Congestive heart failure	12 (0.9)	46 (0.8)	0.611	1.287	0.657–2.523	0.462
Acute heart failure	30 (2.3)	119 (2.0)	0.521	1.244	0.791–1.955	0.344
Stroke	1 (0.1)	3 (0.1)	0.551	–	–	0.996
Pneumonia	0 (0.0)	6 (0.1)	0.600	–	–	0.988

Values are presented as n (%). Unadjusted P values were obtained using chi-square or Fisher’s exact tests, as appropriate. Adjusted odds ratios (aORs) and 95% confidence intervals (CIs) were estimated using multivariable logistic regression adjusted for the covariates listed in Table 1, including calendar year fixed effects (YEAR 2016–2022). “–” indicates that adjusted estimates were not estimable because of sparse events and model nonconvergence; in such cases, the adjusted P value was derived from the Score test and should be interpreted cautiously. Rare events (e.g., stroke and pneumonia) are also summarized descriptively in Supplementary Table S2. RA-UKA, robotic-assisted unicompartmental knee arthroplasty; C-UKA, conventional unicompartmental knee arthroplasty; AKI, acute kidney injury; aOR, adjusted odds ratio; CI, confidence interval

### Surgical and thromboembolic complications

Univariable comparisons showed no statistically significant differences between RA-UKA and C-UKA in pulmonary embolism (*P* = 0.593), deep vein thrombosis (*P* = 0.999), or blood loss anemia (*P* = 0.178) (Table 4).

Table 4 presents adjusted odds ratios for thromboembolic events and blood loss anemia. For pulmonary embolism and deep vein thrombosis, regression models again did not converge because of extremely low event counts, yielding non-estimable adjusted estimates and 95% CIs. The P values from the Score test were 0.983 for pulmonary embolism and 0.993 for deep vein thrombosis, and thus no statistically significant differences in these complications could be demonstrated between RA-UKA and C-UKA.

Blood loss anemia occurred more frequently than the other surgical complications analyzed, permitting estimation of an adjusted odds ratio. RA-UKA was associated with an aOR of 1.222 (95% CI 0.974–1.533; *P* = 0.083) for blood loss anemia compared with C-UKA. Although the point estimate exceeds 1.0, the confidence interval includes unity and the P value does not reach conventional levels of statistical significance, indicating that no significant difference in the odds of blood loss anemia could be established between the two approaches.

**Table 4:** Univariable and multivariable analyses of surgical complications after UKA

Complication	RA-UKA (*n* = 1297)	C-UKA (*n* = 5857)	Unadjusted *P*	Adjusted aOR	95% CI	Adjusted *P*
Pulmonary embolism, n (%)	0 (0.0)	5 (0.1)	0.593	–	–	0.983
DVT, n (%)	0 (0.0)	2 (0.0)	0.999	–	–	0.993
Blood loss anemia, n (%)	107 (8.2)	420 (7.2)	0.178	1.222	0.974–1.533	0.083

Values are presented as n (%). Unadjusted P values were obtained using chi-square or Fisher’s exact tests, as appropriate. Adjusted odds ratios (aORs) and 95% confidence intervals (CIs) were estimated using multivariable logistic regression adjusted for the covariates listed in Table 1, including calendar year fixed effects (YEAR 2016–2022). “–” indicates that adjusted estimates were not estimable because of sparse events and model nonconvergence; in such cases, the adjusted P value was derived from the Score test and should be interpreted cautiously. *RA-UKA* robotic-assisted unicompartmental knee arthroplasty, *C-UKA* conventional unicompartmental knee arthroplasty, *DVT* deep vein thrombosis, *aOR* adjusted odds ratio, *CI* confidence interval

## Discussion

This national inpatient study showed that RA-UKA represented a minority, yet still a substantial proportion, of UKA procedures in a large U.S. inpatient dataset. In our cohort, approximately 18% of procedures were performed with robotic assistance, which is broadly consistent with contemporary estimates from other settings [2]. Hospital teaching status differed between groups, suggesting institutional differences in practice patterns related to the use of robotic assistance for UKA. Evaluating the effects of RA-UKA on clinical outcomes and billed charges in routine practice therefore remains an important priority. Because total hospital charges (TOTCHG) in the NIS represent billed charges recorded in nominal dollars for the discharge year, absolute charge values should be interpreted cautiously across the 2016–2022 study period. Although calendar year was included as a fixed effect in the regression models to partially account for secular trends, inflation adjustment to constant dollars was not performed.

Given the observational design, these findings indicate association rather than causation; institutional pathways, discharge practices, and case selection may have contributed to the observed LOS patterns. In this national inpatient analysis, RA-UKA was associated with lower odds of prolonged hospitalization (LOS ≥ 2 days, corresponding to the 75th percentile) compared with C-UKA. This finding may reflect differences in perioperative pathways, discharge practices, and patient selection at institutions adopting robotics, rather than a direct causal effect of robotic assistance on recovery. Our findings are generally consistent with prior clinical reports. For example, a territory-wide analysis from Hong Kong reported that Mako robotic-assisted UKA had a shorter average length of stay than conventional UKA (2.9 ± 1.6 vs. 3.6 ± 2.6 days), whereas the overall robotic-assisted group showed similar LOS to conventional UKA, suggesting that absolute LOS effects may vary by platform and healthcare system [2]. Potential explanations proposed in the literature include more reproducible bone preparation and soft-tissue handling, which may facilitate earlier mobilization; however, these mechanistic hypotheses cannot be evaluated in the NIS. Accordingly, several studies have reported improved early performance metrics after RA-UKA, suggesting more rapid early functional recovery [21, 22]. A reduction in prolonged inpatient stays may align with enhanced recovery objectives in joint arthroplasty, although effects on patient-reported outcomes and broader system-level efficiency require prospective evaluation.

Both univariable and multivariable analyses showed no significant differences in early medical or thromboembolic complications between RA-UKA and C-UKA during the index hospitalization. Acute adverse events, including venous thromboembolism, acute coronary syndrome, cerebrovascular accident, and pneumonia, were infrequent in both cohorts, and the study had limited power to detect modest differences because of the low event rates. This early safety profile is broadly consistent with prior literature indicating that UKA, as a procedure, is associated with lower perioperative morbidity than TKA [10]. These findings do not suggest that robotic assistance materially alters the in-hospital medical adverse event profile. This interpretation is consistent with prior literature suggesting that, despite improved technical precision with robotic systems, clear evidence for a reduction in early complications remains limited [23]. Our data therefore support the interpretation of comparable short-term inpatient safety between the two approaches.

Because the NIS captures the index hospitalization only, revision risk, implant survivorship, and patient-reported outcomes cannot be evaluated in the present analysis. Although prior clinical literature suggests that robotic assistance may improve component positioning and reduce alignment outliers, and some studies have reported differences in functional outcomes or revision-related endpoints [21], any potential downstream clinical benefits remain outside the scope of inpatient administrative data and should not be inferred from our findings. Accordingly, the present results should be interpreted as reflecting inpatient utilization patterns and perioperative care pathways rather than long-term comparative effectiveness. The very low inpatient mortality observed in this study is not unexpected, given the low fatality rate of UKA and the brief observation window of the NIS. Therefore, questions regarding possible intermediate- or longer-term differences require dedicated longitudinal follow-up beyond the inpatient stay.

The economic implications of RA-UKA remain debated. In our study, RA-UKA was not associated with higher odds of a high-charge admission during the index hospitalization compared with C-UKA. Importantly, TOTCHG represents billed charges rather than true costs, reimbursements, or net financial impact; therefore, our findings should be interpreted as inpatient charge patterns rather than definitive cost-effectiveness. The broader economic value of RA-UKA likely depends on longer-term outcomes not captured in the NIS, including revision-related events, reoperations, rehabilitation utilization, and downstream healthcare use. Prior studies have reported mixed short-term economic findings, with some describing modestly higher perioperative charges or index-admission costs for RA-UKA [24, 25]. Conversely, cost-effectiveness analyses have suggested that RA-UKA could become economically favorable over time under certain assumptions regarding revision burden, quality-adjusted life years, and procedural efficiency, particularly in higher-volume settings [23]. Accordingly, our results inform billed charge patterns during the index hospitalization only and do not resolve the long-term cost-effectiveness of RA-UKA.

Overall, RA-UKA was associated with lower odds of prolonged hospitalization and no higher odds of a high-charge admission during the index hospitalization. Early in-hospital complications were uncommon, and no significant between-group differences were detected. These findings likely reflect a combination of institutional pathways, discharge practices, and case selection and should not be interpreted as causal effects of robotic assistance. Future studies incorporating longitudinal follow-up, surgeon- and hospital-level factors, and patient-reported outcomes are needed to better clarify longer-term comparative effectiveness and economic value.

### Limitations

This study has several limitations. First, its retrospective observational design and reliance on administrative coding introduce the possibility of residual confounding and coding misclassification, including potential undercoding of robotic-assisted procedures. In addition, because laterality and bilateral staging cannot be fully characterized for all admissions in the NIS, inadvertent inclusion of bilateral or staged procedures cannot be completely excluded. Second, the NIS captures only the index hospitalization and does not provide post-discharge outcomes, revisions, patient-reported outcomes, implant details, surgeon volume, or procedure-specific hospital volume. Third, several inpatient complications were rare, limiting statistical power for sparse-event endpoints and resulting in non-estimable adjusted models for some outcomes. Fourth, TOTCHG was analyzed as reported by the NIS in nominal dollars without inflation adjustment, and pre/post-COVID sensitivity analyses were not performed; therefore, some temporal heterogeneity may remain despite adjustment for calendar year fixed effects. Fifth, LOS and TOTCHG were dichotomized at the 75th percentile for the primary adjusted analyses, which may reduce information compared with continuous modeling, although this approach was selected to capture upper-tail utilization while maintaining adequate event counts for stable regression. Finally, analyses were performed on the unweighted discharge-level sample, and cases with missing race were excluded from multivariable models; therefore, the findings reflect associations within the analytic cohort rather than nationally weighted estimates, which may limit national generalizability.

## Conclusion

In this national inpatient cohort, RA-UKA was independently associated with lower odds of prolonged hospitalization and no higher odds of high-charge admission during the index hospitalization. Early in-hospital complications and mortality were uncommon, and no significant between-group differences were detected. These findings apply to the index hospitalization only and should not be extrapolated to long-term outcomes.

## Supplementary Information

Below is the link to the electronic supplementary material.

Supplementary Material 1

## Data Availability

The data that support the findings of this study are available from the Healthcare Cost and Utilization Project (HCUP) Nationwide Inpatient Sample (NIS). Restrictions apply to the availability of these data, which were used under license for the current study and are not publicly available. Data are available from HCUP (AHRQ) upon reasonable request and with permission/licensing.
