# Supplementary material for: Robotic-assisted unicompartmental knee arthroplasty is associated with lower odds of prolonged hospitalization and no higher odds of high-charge admission during the index hospitalization
Source: Arch Orthop Trauma Surg. 2026 May 9;146(1):179. doi: 10.1007/s00402-026-06336-x (PMC13157421; doi:10.1007/s00402-026-06336-x)
Supplement: Supplementary file 1 — Supplementary Material 1 [file 402_2026_6336_MOESM1_ESM.docx]

Table S1. ICD 10 CODES / PROCEDURE CODE

| ICD 10 CODES / PROCEDURE CODE |  |
| --- | --- |
| ICD-10-P-0SRC0L9,ICD-10-P-0SRC0LA,ICD-10-P-0SRC0LZ,ICD-10-P-0SRD0L9,ICD-10-P-0SRD0LA,ICD-10-P-0SRD0LZ | UKA |
| 8E0Y0CZ,8E0YXCZ | Robotic Assisted Procedure of Lower Extremity |
| I5021, I5031, I5033, I5041, I5043 | Acute heart failure |
| N170, N171, N172, N178, N179 | Acute Kidney Injury |
| I2101, I2102, I2109, I211, I2119, I2111, I212, I2129, I213, I214, I219 | Acute Coronary Artery Disease |
| I60, I61, I62, I63, I650, I688, O873, O2250, O2251, O2252 | Stroke |
| J810, J811, I501 | Pulmonary Edema |
| I10(start with) | Hypertension |
| D62 (start with) | Blood Loss Anemia |
| J189, J159, J22 | Pneumonia |
| I2602, I2609, I2692, I2699 | Pulmonary Embolism |
| I82401, I82402, I82403, I82409, I82411, I82412, I82413, I82419, I82421, I82422, I82423, I82429 | DVT |
| E78(start with) | Dyslipidemia |
| G473 | Obstructive Sleep Apnea |
| D64(start with) | Chronic Anemia |
| F10 | Alcohol Abuse History |
| M81, M82 | Osteoporosis |
| F (start with) | Mental Disorders |
| G20 (start with) | Parkinson Disease |
| E11 (start with) | Type 2 Diabetes Mellitus |
| N18 (start with) | Chronic Kidney Disease |
| I500, I501, I509 | Congestive Heart Failure |
| J44 (start with) | Chronic Lung Disease |
|  |  |

Table S2. Rare in-hospital events with sparse counts

| Outcome | RA-UKA (n) | C-UKA (n) |
| --- | --- | --- |
| Stroke | 1 | 3 |
| Pneumonia | 0 | 6 |
| In-hospital mortality | 0 | 1 |

Note: Values are presented as the number of events. Because event counts were extremely low (including zero cells), regression-based adjusted estimates may be unstable or non-estimable; therefore, these outcomes are summarized descriptively.
